# Flotillins Are Involved in the Polarization of Primitive and Mature Hematopoietic Cells

**DOI:** 10.1371/journal.pone.0008290

**Published:** 2009-12-22

**Authors:** Lawrence Rajendran, Julia Beckmann, Astrid Magenau, Eva-Maria Boneberg, Katharina Gaus, Antonella Viola, Bernd Giebel, Harald Illges

**Affiliations:** 1 Systems and Cell Biology of Neurodegeneration, Department of Psychiatry Research, University of Zurich, Zurich, Switzerland; 2 Immunology, University of Konstanz, Konstanz, Germany; 3 Max-Planck Institute of Molecular Cell Biology and Genetics, Dresden, Germany; 4 Institute for Transplantation Diagnostics and Cellular Therapeutics, Heinrich-Heine-University Düsseldorf, Düsseldorf, Germany; 5 Institute of Transfusionsmedicine, Transplantations Diagnostics, Universitätsklinikum Essen, Essen, Germany; 6 Centre for Vascular Research, University of New South Wales, Sydney, Australia; 7 Department of Haematology, Prince of Wales Hospital, Sydney, Australia; 8 Biotechnologie Institut Thurgau, Taegerwilen, Switzerland; 9 Department of Biomedical Sciences, University of Padova, Padova, Italy; 10 Immunology and Cell Biology, University of Applied Sciences, Rheinbach, Germany; Fundação Oswaldo Cruz, Brazil

## Abstract

**Background:**

Migration of mature and immature leukocytes in response to chemokines is not only essential during inflammation and host defense, but also during development of the hematopoietic system. Many molecules implicated in migratory polarity show uniform cellular distribution under non-activated conditions, but acquire a polarized localization upon exposure to migratory cues.

**Methodology/Principal Findings:**

Here, we present evidence that raft-associated endocytic proteins (flotillins) are pre-assembled in lymphoid, myeloid and primitive hematopoietic cells and accumulate in the uropod during migration. Furthermore, flotillins display a polarized distribution during immunological synapse formation. Employing the membrane lipid-order sensitive probe Laurdan, we show that flotillin accumulation in the immunological synapse is concomittant with membrane ordering in these regions.

**Conclusions:**

Together with the observation that flotillin polarization does not occur in other polarized cell types such as polarized epithelial cells, our results suggest a specific role for flotillins in hematopoietic cell polarization. Based on our results, we propose that in hematopoietic cells, flotillins provide intrinsic cues that govern segregation of certain microdomain-associated molecules during immune cell polarization.

## Introduction

Establishment of cell polarity is crucial for the migration of immature and mature leukocytes across vascular endothelial barriers and provides the basis for hematopoietic stem and progenitor cell (HSC/HPC) homing [1], [2]. Directional cues for the migration of leukocytes to the sites of inflammation are provided by the chemokine family members [3] and are promptly executed via intrinsic signaling pathways downstream of chemokine receptors. Similarly, chemokines have been implicated in controlling HSC/HPC homing [1]. In both contexts, cells switch from spherical to polarized phenotypes in response to polarization cues. These morphological changes are accompanied by an orchestrated compartmentalization of certain cell surface associated molecules [4], [5], [6]. Additionally, cytoskeletal reorganization [2], [7], lateral compartmentalization of functional membrane microdomains [6], [8] and redistribution of some cellular proteins have been observed [9]. Actin cytoskeletal rearrangement during directional migration is a highly conserved and well documented process in amoeboid cells [10], [11], [12], and leukocytes display the characteristic leading and trailing edges [2]. While the leading edge is marked by a concentration of F-actin [9], [10] and chemokine receptors [8], [12], [13], the trailing edge, termed uropod, is marked by the accumulation of several adhesion molecules [4], [6], [14], the hyaluron receptor [15], [16], sialoglycoproteins [4], [17], [18], and the ERM (Ezrin, Radixin and Moesin) family of proteins [2]. Lipids also show different polarization patterns during lymphocyte migration [6], [8]. Along similar lines, insights into the importance of lipid rafts or membrane microdomains in the process of chemokine-induced polarization have been provided by recent studies [8], [17], [19], [20]. However, none of the molecules implicated in either chemokine-induced polarization or raft residency show asymmetric localization under resting conditions that could impart pre-polarization cues. We recently showed that the lipid microdomain resident proteins, flotillin-1 and -2 [21], [22], confer intrinsic polarity to leukocytes by their asymmetric localization [23]. In the current study, we show that flotillin-1 and -2 accumulate at uropods and co-localize with CD43, CD44, and moesin, a member of the ERM family and present evidence for the distinct spatial and temporal localization of flotillin platforms with respect to the actin cytoskeleton upon chemokine-induced migration. This polarization of flotillins appears specific for hematopoietic cells, as it is not observed in other, non-hematopoietic polarized cell types. Moreover, flotillins accumulated at the central supramolecular activation cluster (c-SMAC) during immunological synapse formation concomitant with membrane ordering in these regions. Based on our results, we propose that a subset of lipid microdomains provide pre-polarization cues for hematopoietic cell polarization.

## Results

A key issue in hematopoietic cell biology is the mechanism by which cell polarity is initiated. Do leukocytes prearrange certain molecules and machineries in a polarized fashion prior to the acquisition of polarized morphologies, or are extrinsic cues alone responsible to induce all events required for cellular polarization? Since chemokine induced polarization of leukocytes and synapse formation between lymphocytes typically occurs within seconds, existence of intrinsic cues for polarization cannot be ruled out. We previously showed that a family of membrane microdomain-associated molecules called flotillins [21], [22] are asymmetrically localized in various non-activated hematopoeitic cells and hypothesized a possible involvement of this feature in imparting intrinsic polarity [23]. Here we investigated the pre-assembled flotillin platforms in the light of chemokine induced migration of T- cells, monocytes and hematopoietic stem cells/progenitor cells (HSC/HPC) and polarization during immunological synapse formation.

### Chemokine Treatment Induces Cytoskeletal Reorganization in T Lymphocytes

**Figure 1:**
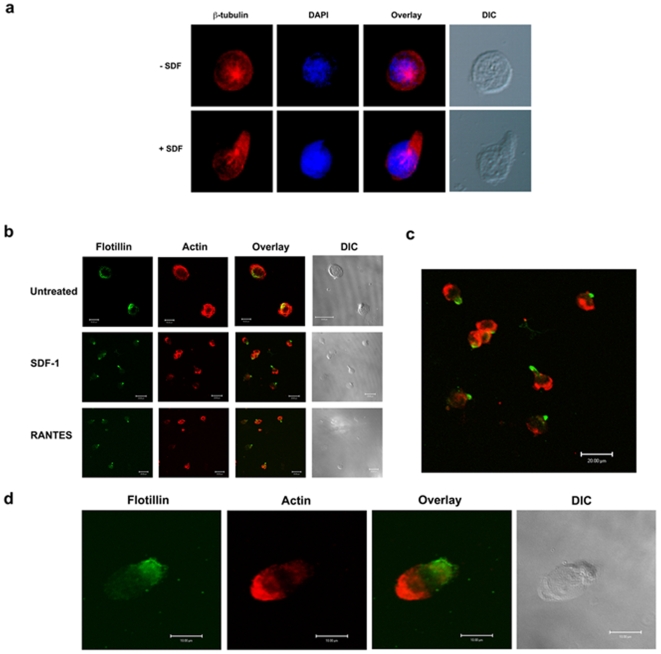
Pre-assembled platforms of flotillins polarize to the uropods upon chemokinesis and the spatial segregation of flotillins and actin cytoskeleton during polarization. a) T lymphoblasts were treated with SDF-1α and allowed to migrate on fibronectin. The control cells were just plated on fibronectin without any chemoattractant. The cells were then fixed and stained with β-tubulin (red) and DAPI (blue) to stain the microtubules and the nucleus, respectively. Note that during chemokinesis, the microtubules and the MTOCs reorient themselves to the rear end of the cell. b) Untreated T lymphoblasts show uniform actin distribution but very polarized flotillin-1 staining (green). But chemoattracted (SDF-1α or RANTES) treated cells show polarized morphology accumulating actin (red) at the leading edge and flotillins (green) at the uropod. The images represent several sets of experiments and images collected. c) Magnified image of an SDF-1α treated lymphocyte displaying a clear spatial distribution of flotillins and actin cytoskeleton. d) Note that, even before a morphologic polarization took place, flotillins and actin spatially distribute themselves as early as 5 min after stimulation.

To study cell polarity, we stimulated T lymphoblasts with chemokines and allowed them to migrate on fibronectin. Non-stimulated T lymphoblasts are morphologically round with β-tubulin at the perinuclear compartment (Fig. 1a). Upon stimulation with the chemokine SDF-1α, the lymphoblasts underwent drastic morphological changes and acquired a polarized cell shape displaying leading edge and uropod regions with β-tubulin staining observed now more prominently at the base of uropod regions (Fig. 1a). Comparable results were obtained when cells were stimulated with RANTES (data not shown).

### Chemokine-Induced Polarization Redistributes Flotillins to the Uropod and Actin to the Leading Edge

In order to study the localization of flotillins during chemokine-induced polarization, we employed confocal imaging on T-lymphoblasts that were immunostained for flotillin and actin to mark the leading edge. As shown in Fig. 1b, the unstimulated lymphoblasts showed uniform actin distribution but displayed a polarized flotillin staining. In stimulated cells, flotillin staining was co-incident with the uropod regions of the lymphoblast whereas actin accumulated at the leading edge, leading to spatial segregation of these two components (Fig. 1b, c). Both chemokines (SDF-1 and RANTES) induced similar segregation of flotillins away from the actin cytoskeleton during chemokinesis.

Reorganization of the actin cytoskeleton during migration is an essential process involving concerted action of several actin binding proteins and the exclusion of adhesion related molecules to the rear end [8], [10]. A clear spatial separation of actin and flotillins occurred as early as 5 minutes after stimulation (Fig. 1d), before any morphological separation of leading and trailing edges became apparent. Upon prolonged activation (30 min), flotillins were distributed to the morphologically defined uropods and the actin cytoskeleton to the leading edges.

### Flotillins Co-Localize with Known Uropod Markers upon Lymphocyte Polarization

**Figure 2:**
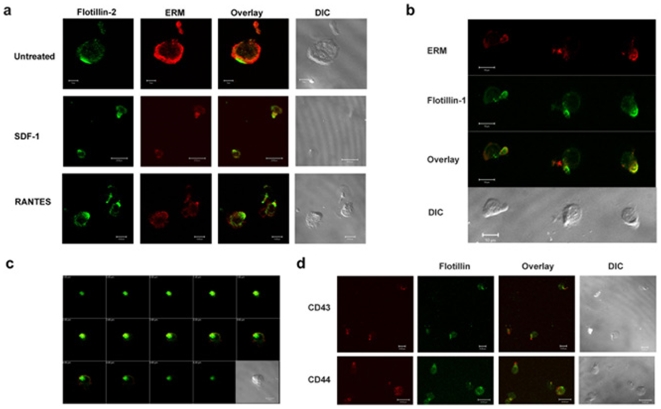
Flotillins co-localize with uropod markers upon chemokinesis in T lymphoblasts. a) SDF-1α or RANTES treated lymphoblasts were stained with anti-flotillin-2 (green) and anti-ERM (red) antibodies. DIC images show morphological polarization during migration. b) ERM (red) and flotillin-1 (green) stained RANTES treated lymphoblast show that both the molecules concentrate at the uropod during migration. Note that ERM staining is also found at the leading edge due to the fact that the antibody also recognizes ezrin, which is localized at the leading edge. c) Peripheral T lymphoblasts were treated with RANTES, fixed and stained for flotillins (green) and ERM (red) proteins. Series of Z-stack images show that flotillins are more contained to the uropods. d) Chemokine stimulated cells were allowed to migrate on fibronectin and stained with anti-CD43 (upper panel, red), CD44 (lower panel, red) and anti-flotillin antibodies (green). The DIC images show the contrast image of the polarized lymphoblasts.

To address the question whether flotillin platforms co-localized with known uropod markers, we stimulated T lymphoblasts with SDF-1α or RANTES, allowed them to migrate on fibronectin and immunostained for the uropod markers. The band 4.1 family proteins such as Ezrin, Radixin and Moesin (ERM) were recently characterized to be redistributed to uropods upon chemokine induced polarization [4], [9]. Double immunostaining with anti-ERM antibodies and anti-flotillin-1 or –2 antibodies showed that whilst flotillins were polarized in pre-assembled clusters before stimulation, ERM proteins were uniformly distributed at the cell surface (Fig. 2a). Upon chemokinesis, ERM proteins distributed mainly to the uropod as indicated by strong immunostaining at the rear end, where they co-localized with flotillin-1 and -2 (Fig. 2a–c). In addition, in our co-localization experiments, uropod-located flotillins co-localized with adhesion molecules such as CD43 and CD44 (Fig. 2d) confirming flotillins polarization to uropods during T cell migration.

### Polarity of Flotillins in Migrating HSC/HPC

**Figure 3:**
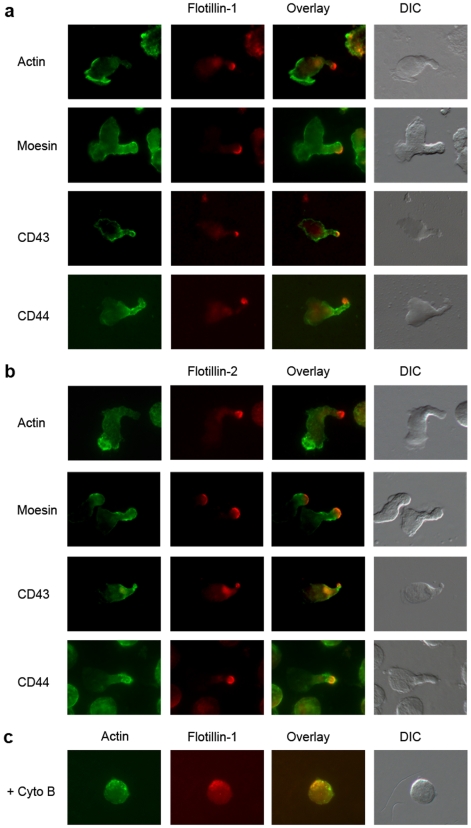
Flotillin polarization to the uropods in cultivated CD34^+^ cells. In morphological polarized CD34^+^ cells flotillin-1 (a) and flotillin-2 (b) are highly concentrated in the tip of the uropod while moesin, CD43 and CD44 are located in the whole uropod. Sometimes an additional domain of flotillin-2 is found at the base of the uropod (arrow). Actin marks the leading edge. (c) Cyto B panel shows the morphology and the staining of actin and flotillin-1 in cytochalasin B treated cells.

To determine whether the polarized localization of flotillins to T cell uropods is specific to T cells or a general feature of migrating hematopoietic cells, we studied flotillin distribution in primitive hematopoietic cells (CB-derived CD34^+^ cells) and myeloid cells (chemokine-induced monocytes). Recently, we showed that upon cultivation, CD34^+^ hematopoietic cells polarize and establish a leading and a trailing edge [6]. To analyze flotillin polarization in these cells, we studied flotillin distribution in relation to the uropod antigens moesin, CD43 and CD44, as well as to actin. Microscopic analysis revealed that both flotillin-1 and -2 co-localize at the tip of the uropods with the ERM protein, moesin, along with CD43 and CD44 while segregating away from the leading edge marker, actin (Fig. 3). Live cell imaging of flotillin-2-GFP transfected CD34^+^ hematopoietic cells showed that flotillin-2 remained polarized to the uropods during migration (Video S1). Destabilization of actin led to remarked loss in the cell shape and consequently loss in polarized flotillin localization (Fig. 3). These results suggest that, as in polarized T cells, flotillins are asymmetrically localized in HSC/HPC to uropod-like domains during their polarization and that actin is required for the migratory polarity.

### Polarity of Flotillins in GM-CSF Polarized Monocytes

**Figure 4:**
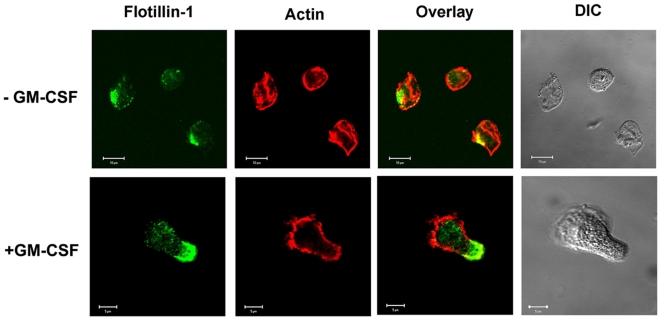
Spatial distribution of flotillins to the uropods and actin to the leading edge during GM-CSF induced monocyte polarization. GM-CSF untreated (upper panel) and treated (lower panel) macrophages were stained with anti-actin (red) and anti-flotillin-1 (green).

The asymmetric distribution of flotillin in hematopoietic progenitors led us to ask whether flotillin polarization also exists in cells of myeloid origin. Since the chemokine GM-CSF has been found to induce migration of monocytes and macrophages, [24] we treated both cell types with GM-CSF. As observed in T lymphoblasts, unstimulated peripheral blood monocytes showed evenly distributed actin staining but exhibited polarized flotillin-1 localization (Fig. 4, upper panel). GM-CSF treatment not only induced a migratory phenotype in monocytes but also showed an intense actin staining at the leading edge. Comparable to stimulated T-lymphoblasts and the cultivated CD34^+^ cells, flotillins were enriched at the tip of the uropods of polarized monocytes (Fig. 4, lower panel). Flotillin-1 and -2 also co-localized with uropod markers (data not shown). Together, these results suggest that upon adaptation of migratory polarity, flotillins specifically become localized in the uropod, both in immature and mature hematopoietic cells.

### Chemokine Stimulation Induces Co-Localization of Flotillins with GM1 Rafts but Does Not Affect the Raft Residency

**Figure 5:**
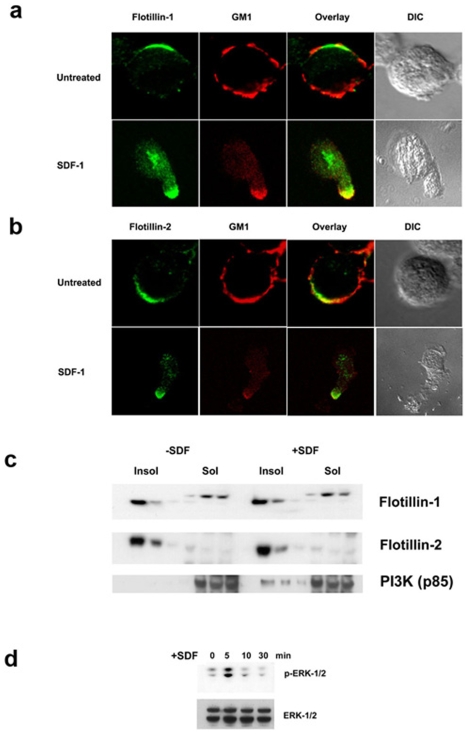
SDF-1α treatment induces reorganization of GM1- and flotillin-microdomains but does not affect DRM localization of flotillins in T cells. a) Unstimulated (upper panel) and SDF-1a treated (lower panel) cells were stained with mouse anti-flotillin-1 (green) to detect flotillin-1. To detect the ganglioside, GM1, cells were stained first with Cholera toxin – B (CTX-B) subunit and then with rabbit anti- CTX-B antibodies (red). b) Unstimulated (upper panel) and SDF-1a treated (lower panel) cells were stained with mouse anti-flotillin-2 (green) to detect flotillin-2. GM1, on the other hand is detected by staining the cells first with Cholera toxin – B (CTX-B) subunit and then with rabbit anti- CTX-B antibodies (red). c) DRM fractions isolated from control (left) and SDF-1α treated (right) T cells were blotted for flotillin-1 (upper panel), flotillin-2 (middle panel) and PI3K (lower panel). Note that microdomain residency of flotillins is not affected during the treatment but PI3K specifically translocates to DRMs only upon chemokine stimulation. d) Whole cell lysates of SDF-1α treated cells for various times as indicated were boiled, electrophoresed and transferred onto a nitrocellulose membrane. *Upper panel*: The membrane was then probed with anti-phospho-ERK1/2 antibodies. Increased ERK phosphorylation accompanied chemokine treatment at 5 min and decreases with time. *Lower panel*: Blotting with anti-ERK antibody shows the total ERK content.

The uropod and the leading edge are not only morphologically identifiable, but are also biochemically distinct [8], [13]. While the leading edge harbors proteins such as chemokine receptors and F-actin, the uropod is enriched in adhesion molecules [6], [25]. Apart from proteins, certain lipids are also enriched in these domains. Particularly, the ganglioside GM1 is highly concentrated in uropods while the ganglioside GM3 is enriched in leading edges [6], [8]. In order to further characterize the uropod localization of flotillins, we stained T lymphocytes for GM1 and flotillin. As shown in Fig. 5, non-stimulated T cells exhibited an asymmetric distribution of both flotillin-1 (Fig. 5a) and flotillin-2 (Fig. 5b) while GM1, visualized by Cholera-Toxin subunit B (CTX-B) staining, did not show this striking polarization. Upon SDF-1α treatment, flotillins were enriched at the uropods that also concentrated almost all of the cellular GM1 (Fig. 5a, 5b).

Since flotillins were polarized before and after polarization, we decided to test whether raft association of flotillins was also preserved upon stimulation. Preparation of detergent resistant membrane (DRM) fractions, as a means of isolating raft-like domains, clearly showed that flotillins were mainly localized to the DRM fractions (Fig. 5c), both before and after stimulation. On the other hand, signaling molecules such as p85 PI3K, the regulatory subunit of phosphoinositol-3-kinase, remained soluble in the non-activated conditions and translocated to the DRMs upon SDF-1 treatment (Fig. 5c). As DRMs cannot distinguish L-rafts (leading edge rafts) and U-rafts (uropod rafts), our data suggest that flotillins are constitutive components of lipid rafts while other molecules such as the PI3K require chemotactic stimulation for recruitment to these signaling platforms. These results correspond to our microscopy studies (Fig. 1 and 2) showing that the flotillins exist as visible and stable pre-assembled platforms before and after stimulation.

In order to confirm that the chemokine treatment indeed stimulated the cells, SDF-1α-treated lysates were blotted and probed for ERK activation [26]. An increase in ERK phosphorylation (Fig. 5d, upper panel) accompanied SDF-1α treatment without affecting the total ERK levels (Fig. 5d, lower panel). This result implied that chemokine stimulation induced activation of cells via an ERK-dependent signaling pathway, while the raft association of flotillins remained unchanged during chemokine-induced polarization of leukocytes.

### Polarization of Flotillins during Immunological Synapse (IS) Formation

**Figure 6:**
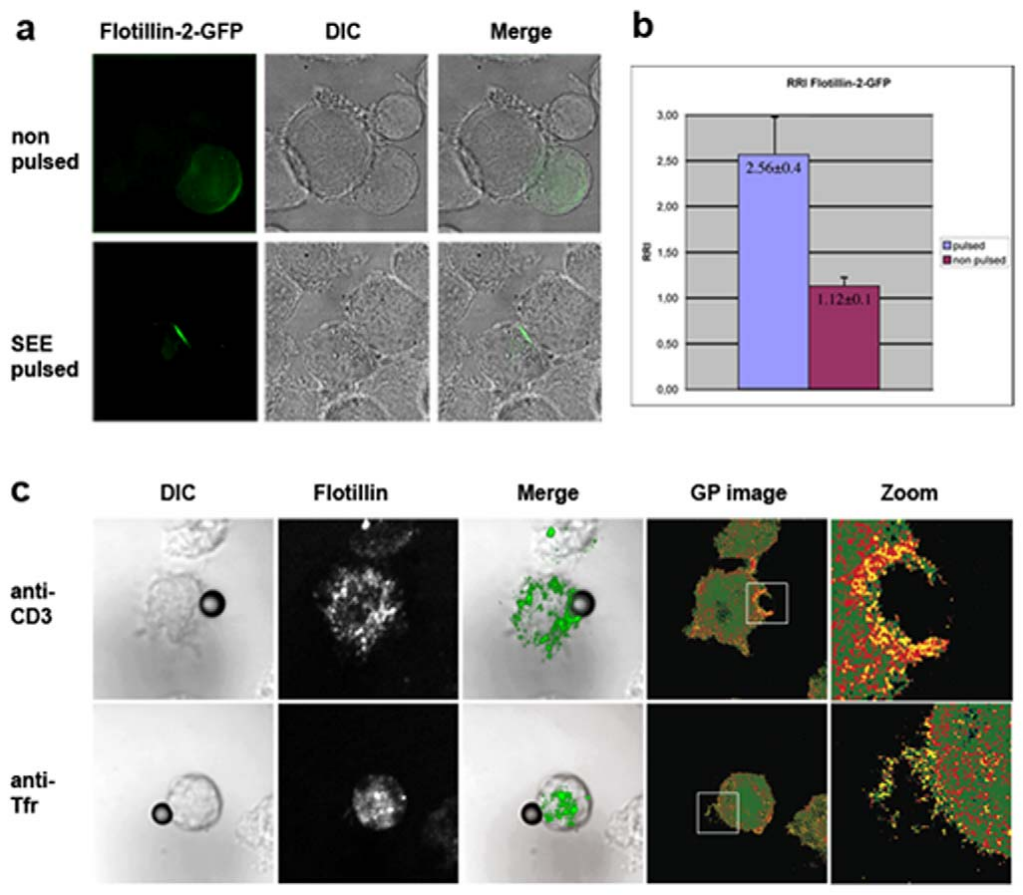
Flotillin polarization to the immunological synapse is concomitant with membrane condensation. a) Jurkat cells stably expressing Flotillin-2-GFP were incubated with either unpulsed or SEE-pulsed EBV-B cells for 20 min. Confocal images were taken with identical settings. b) Quantitation of the conjugate formation in non-pulsed and SEE-pulsed conditions. c) Jurkat cells were labeled with Laurdan, conjugated with anti-CD3 antibody- or anti-TfR antibody-coated beads on ice and activated for 10 min at 37°C. After fixation, cells were immunostained for flotillin-1, adhered to poly-L-lysine-coated coverslips, mounted and imaged. The confocal image of flotillin-1 is recorded at the identical focal depth as the Laurdan images that are converted into GP images as described in *Methods.* GP images are pseudocolored with high GP (ordered membranes) in yellow and low GP (fluid membranes) in green.

Having shown that flotillins polarized to the uropods during chemotactic migration, we studied their polarization during other activation cues. Similar to chemotaxis, T lymphocyte activation requires asymmetric redistribution of membrane receptors, signaling molecules and the actin cytoskeleton [27]. T lymphocyte activation is a consequence of the interaction between T cell receptors (TCRs) and specific antigenic complexes formed by antigen-derived peptides bound to integral membrane proteins encoded by the class I or class II genes of the major histocompatibility complex (MHC)[27], [28]. During this activation, the triggered TCRs and their signaling partners are spatially organized in supra-molecular signaling complexes and this spatial organization plays a central role in the initiation, modulation and probably termination of signaling [5], [27], [28]. These complex and dynamic structures at the interface between T cells and antigen-presenting cells are termed “immunological synapses” (IS)[28]. Lipid rafts appear to be key organizing elements of T cell IS [29]. Since flotillins polarized to uropods during migration, we speculated that flotillin may be specifically recruited into IS during T cell activation. Thus, we analyzed by confocal microscopy the distribution of Flotillin-2-GFP in Jurkat T cells incubated with SEE-pulsed EBV-B cells. Although unpulsed cells showed pre-polarization of flotillin, we found that Flotillin-2-GFP accumulated at the IS only when T cells were incubated with pulsed B cells, that is, only when TCR triggering had induced IS formation and T cell polarization (Fig. 6a). These results clearly showed that not only migratory cues but also antigen presentation induced flotillin polarization.

### Flotillins Localize to the Highly Ordered Regions at the Synapse

To address whether flotillins associate with raft domains at activations sites, we employed Laurdan, a fluorescent membrane order dye [24], [25] which exhibits a 50-nm red shift as membranes undergo transition from disordered to liquid-ordered [24], [25]. By calculating a normalized intensity ratio in two simultaneously recorded spectral channels, defined as Generalized Polarization (GP), we can measure membrane order. To specifically measure membrane order in T cells without contributions from the adjacent APC, we stimulated Jurkat T cells with beads coated with antibodies against CD3 triggering the TCR, or with transferrin receptor (TfR) coated beads as negative controls. As previously reported [25], we found a robust condensation of the membranes at the site of CD3 conjugation as indicated by the high GP values (yellow coloring in Fig. 6b), but not TfR crosslinking (green coloring in Fig. 6b). This membrane condensation coincided with flotillin-1 recruitment to the site of TCR stimulation, while flotillin-1 was not detected at the contact site to anti-TfR-coated beads. These results show that raft-associated flotillins become enriched at immunological synapses.

### Absence of Flotillin Polarization in Polarized Epithelial Cells

**Figure 7:**
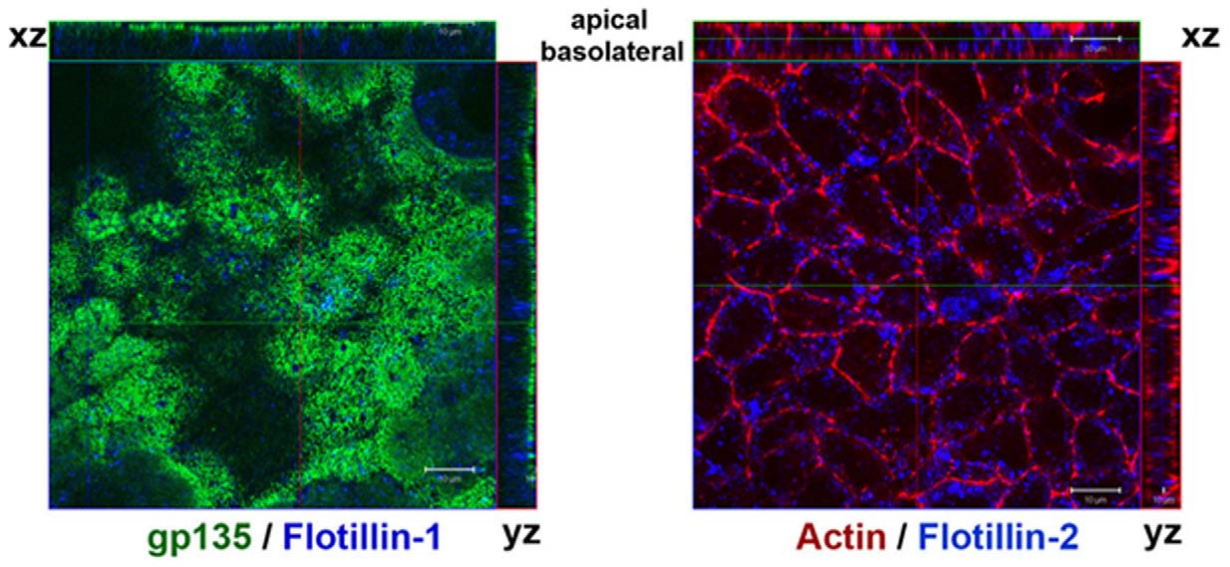
Flotillins are not polarized in MDCK cells. Filter-grown MDCK cells were immunostained for apical marker protein, GP135 (green) and flotillin-1 (blue) or with Phalloidin to stain actin (red) and flotillin-2 (blue). Note that GP135 marks exclusively the apical membrane while flotillin-1 and -2 are mainly associated with intracellular vesicles. Actin, on the other hand marks both apical and basolateral membranes.

Since the results presented thus far showed that flotillins polarize to specific structures during immune cell polarization, we tested whether polarization of flotillins is also observed in cells of non-hematopoietic origin that display polarity. Non-hematopoietic polarized cells include budding yeast, epithelial cells, neural cells, etc [30], and here we studied the localization of flotillins in polarized kidney epithelial cells (MDCK), a model cell line of epithelial polarity [31]. These cells display distinct apical and basolateral domains, which differ distinctly in their lipid and protein compositions. Raft lipids and proteins are sorted predominantly to the apical domain of MDCK cells whereas non-raft cargo is sorted to the basolateral domains [32]. Since, flotillins are *bona fide* raft markers, we examined whether they were polarized to the raft-enriched apical domain that is the epithelial analog of the lymphoid uropods [19], [30]. In filter-grown, polarized MDCK cells, both flotillin-1 and flotillin-2 showed a non-polarized distribution whereas Gp135, a prominent apical protein [26], exhibited a distinct polarized apical localization (Fig. 7). As shown with actin and Gp135 staining, and also with proper localization of ZO-1, a tight junction marker (data not shown), these cells were indeed polarized but failed to exhibit any polarization of flotillins. Moreover, no flotillin polarization was observed during wound healing in these cells (data not shown). This lack of polarized localization of flotillins in other polarization systems suggests that flotillin polarization is specific for hematopoietic cells.

## Discussion

In this work, we show that flotillins exist as preassembled structures under resting conditions in mature and immature hematopoietic cells and polarize to either uropods during cell migration or to the immunological synapse during T cell activation. We additionally present evidence that this feature is specific to cells of hematopoietic origin.

Flotillins belong to a family of proteins that share an evolutionarily conserved stomatin/prohibitin/flotillin/HflK/C (SPFH) domain [21], [22], [33]. Flotillins and stomatins have been shown to be expressed in hematopoietic cells and to be raft associated [21], [34], [35]. We previously showed that flotillins are asymmetrically localized in various hematopoietic cells and that they play a role in T cell activation [23], [36]. Here, we show that this asymmetric localization of flotillins occurs in migratory polarity and during immunological synapse formation, probably imparting intrinsic cues for hematopoietic cell polarization. Views on whether polarity is established only towards extrinsic directional cues or it resides intrinsically in the cell have been ambiguous [11], [37]. Signaling [12] and membrane [38] compartmentalization during cell migration and the ability of cells to respond rapidly to directional cues point to the possible existence of innate mechanisms to compartmentalize certain machinery necessary for the migration and polarization [11]. Intrinsic asymmetry of cytoskeletal and associated molecules in certain parts of the cell might assist in a rapid response to the external stimulus. Several observations support the existence of intrinsic polarity. In *Drosophila* neuroblasts, a well-analyzed cell polarization system, asymmetric distribution of cell fate determinants such as Prospero and Numb is mediated by the activity of PAR complex proteins [39], [40]. In m ammalian lymphocytes, use of *in vivo* three dimensional fluorescence microscopy has shown that effective calcium flux during the immunological synapse (IS) formation occurred only at specific contact sites implicating a possible intrinsic polarity in these cels [41]. However, no direct evidence for intrinsic asymmetry at a molecular level has been shown thus far. From our study we propose that cells of hematopoietic origin do possess intrinsic asymmetry of at least a subset of raft-associated proteins, in particular, flotillins.

Raft-associated proteins have been previously documented to translocate to uropod-like domains upon migration, as well as to the immune synapse during T cell activation [27], [29], [42]. Thus, if one considers flotillins as raft-markers, it might not be surprising to find them in these domains. However, our studies show that flotillins, in contrast to membrane rafts, are preassembled prior to polarization and that could potentially contribute to the formation of uropods or IS upon extrinsic cues. If flotillin polarization during cell migration and activation were the mere consequence of raft coalescence, one would expect them to be enriched in the apical membrane of MDCK epithelial cells, as this compartment is a coalesced raft domain [43]. However, lack of any observable flotillin polarization in these cells rules out that flotillins are “passive” raft markers and suggests that they have a functional role in hematopoeitic cell polarization. The results presented here suggest that pre-assembled flotillin domains could act as a scaffold wherein rafts coalesce, and thereby contribute to the formation and maintenance of uropods. Similar observations of flotillin localization to uropods during polarization of neutrophils were recently reported [44]. In this study, the authors report that in neutrophils, flotillins redistribute to uropods in a polarized manner only after stimulation with chemoattractant and that flotillins interact with PSGL-1 (P-selecting glycoprotein ligand -1) during polarization. Further work is needed to conclusiverly show if pre-assembled regions of flotillin are the sites of uropod formation. Several studies show that a complex restructuring of the leukocyte membrane takes place during migration enabling certain proteins to acquire raft affinity during polarization. Since raft association of flotillins remains unchanged during polarization process, these results show that the microenvironment in which flotillins reside is preserved during polarization while other signaling proteins such as PI3K undergo translocation to raft domains [13]. We believe that raft association of flotillins is essential for its uropod localization, as uropods are enriched in raft lipids [9]. Since most hematopoeitic cells do not contain caveolins, flotillins could serve as raft scaffolding proteins [23], [45], [46]. Considerable amount of evidence suggest that the uropod is a scaffold, assembling several adhesion proteins such as ICAMs, CD43, CD44 and selectins [6], [29], [47], [48]. The uropod enables cell-cell interaction through its resident adhesion molecules and thereby recruits leukocytes to the site of infection [49]. These studies indicate that the uropod serves as an important adhesive scaffold [30]. Occasionally, we also observed clusters of cells, predominantly clustered through their uropod regions, to be enriched in flotillin staining (unpublished observations), probably due to the flotillin interaction with PSGL-1 [44]. These results suggest that in resting cells, preassembled flotillin platforms serve as guidepost for the coalescence of uropod raft components thereby aiding in the uropod assembly.

How do flotillins acquire their asymmetry in resting cells? We have previously shown that flotillin polarization is acquired during cell division [23]. During cell division in *C. elegans* and neurogenesis in *Drosophila*, cell fate determinants are asymmetrically distributed and maintained. Recent work shows that proteins that control asymmetric localization of cell fate determinants also control T cell polarity suggesting conserved mechanisms of polarization [30]. Moreover, antigen stimulated, mitotic T cells have been shown to asymmetrically distribute proteins [30]. Hence it is attractive to speculate that flotillins acquire their polarization during cell division and also maintain their polarized distribution thereafter. Basal PI3K activity (probably maintained through the serum factors) might also be essential for maintaining flotillin polarization in resting state as inhibition of PI3K activity abolishes the asymmetric localization of flotillins and chemokine-induced PI3K activity by establishing cytoskeletal rearrangements might also aid in stabilizing uropod localization of flotillins during polarization. Interestingly, treatment with cytochalasin D or Latrunculin B did not affect the asymmetric localization of flotillins in unstimulated cells suggesting that actin cytoskeleton does not play a major in maintaining the polarity of flotillins. (data not shown). However, actin destabilization clearly affected migration of CD34 positive cells and consequently the localization of flotillins to the uropod in the migrating cells. While actin is not required to maintain the intrinsic polarity of flotillins, migration induced uropod formation requires actin assembly. Whether pre-assembled polarized flotillin platforms give rise to uropods upon migratory cues or migratory cues enable floitllins to localize to newly formed uropods is still to be understood.

Additional mechanisms such as polarized endocytosis and exocytosis could also explain the maintenance of uropod localization of flotillins and other proteins. Polarized delivery of raft-associated proteins directly to uropods has been shown to contribute to the polarity observed in migrating lymphocyte [19]. Not only exocytosis, but also endocytosis of proteins occurs at the uropod thereby accounting for the polarized localization of raft-associated proteins at the uropod region [50]. Recent work shows that polarity is coupled with endo/exocytosis as a homeostatic mechanism to maintain the polarized localization of certain proteins [51]. Given the new role of flotillins in raft-mediated endocytosis [52], [53], [54], [55], [56], it is tempting to propose that flotillins act as scaffolding proteins at the uropod mediating the endocytosis of raft associated molecules and contribute to this homeostasis. Indeed inhibition of endocytosis dramatically reduced both flotillin polarity and migratory polarity (our unpublished observations). Elucidation of the exact mechanism by which pre-assembled flotillin platforms recruit certain raft associated proteins during activation, migration and signaling could provide insights into the raft-clustering induced polarization of leucocytes.

Since disturbances in membrane domain organization and membrane lipid homeostasis affect migration and homing [8], [13], study of mechanisms that control polarization could lend strategies for therapy. Function blocking antibodies of CD44, a uropod-associated protein, inhibited the homing capacity of HSC/HPC and that of AML and CML cells [57], [58], [59], [60], [61], and reduce metastasis of these cells [60], [61]. In the same way, it is possible that interfering with flotillin polarization might affect uropod formation and be a way to reduce metastasis but achieving specificity could be a problem. Indeed high flotillin-2 expression has been shown to be associated with lymph node metastasis and melanoma progression [62], [63] and expression of flotillin-1 and -2 was induced during G-CSF induced myeloid progenitor cell proliferation and granulocyte differentiation [64]. 5′-flanking promoter region of flotillin-1 has shown to contain putative binding sequences for such as myeloid-specific transcription factors AML-1a and MZF-1/2A [65]. These findings encourage a rigorous investigation of the role of flotillins in leukemia, specifically whether reduction in flotillin polarization reduces malignancy. As migratory polarity is intrinsically coupled with metastasis, one might expect that proteins/genes involved in polarization are upregulated during metastasis and targeting polarization processes might be of therapeutic interest.

## Materials and Methods

### Cells and Reagents

Human peripheral T lymphocytes were isolated essentially as described [14]. Human T lymphoblasts were prepared by activating the T lymphocytes on anti-CD3 coated plates for 4-5 days. SDF-1α was purchased from Peprotech (London, UK). RANTES was from R & D, Germany. FN80 coated culture slides and monoclonal antibodies against p85a-PI3K, Flotillin-1 and –2 were purchased from BD Biosciences (Heidelberg, Germany). Antibodies against the ERM proteins and phosphorylated forms of ERK -1 and –2 were from Cell Signal Technology, Frankfurt am Main, Germany. Polyclonal antibodies against WASP, CD44 and CD43 were from Santa Cruz Biotechnology (Heidelberg, Germany). The fluorescent conjugated secondary antibodies were from either Jackson Laboratories (Dianova, Hamburg, Germany) or Molecular Probes (Mobitec, Goettingen, Germany). CTX-B and anti-CTX-B antibodies are from Sigma (Taufkirchen, Germany). Anti-CD14 conjugates with FITC were purchased from Serotec, Oxford, Great Britain. The mounting medium, Prolong™ was from Molecular Probes (Mobitec, Goettingen, Germany).

### Chemokine Induced Migration Studies

T lymphoblasts were plated on 80 kD fragment of Fibronectin (FN80) coated culture slides for 5, 30 or 60 min in the presence or absence of the chemokines, SDF-1α or RANTES at 37°C. SDF-1α was used in the concentration of 100 ng/ml and RANTES, 10 ng/ml. After the indicated periods of incubation, the cells were fixed with ice-cold methanol at −20°C for 5 min or 4% PFA for 10 min at room temperature and processed for confocal microscopy.

### Isolation of CD34^+^ Cells

Human umbilical cord blood (CB) samples were obtained from unrelated donors and are used after informed consent (written) of the mothers according to the declaration of Helsinki.The usage of these materials was approved by the ethics commission of the Heinrich-Heine-University Dusseldorf. Mononuclear cells were isolated from individual samples by Ficoll (Biocoll Separating Solution, Biochrom AG, Berlin Germany) density gradient centrifugation. Remaining red blood cells were lysed at 4°C in 0.83% ammonium chloride with 0.1% potassium hydrogen carbonate, followed by a PBS washing step. CD34^+^ cells were isolated by magnetic cell separation using the MidiMacs technique according to the manufacturer's instructions (Miltenyi Biotec, Bergisch Gladbach, Germany).

### Immunostaining and Microscopy of CD34^+^ Cells

CD34^+^ cells were cultured for 2 days in the presence of FLT3L, SCF, and TPO each at final concentration of 10 ng/mL in IMDM/20% FCS before immunostaining. To conserve their polarized morphology, cultured CD34^+^ cells were fixed for 5 min at room temperature with 1% paraformaldehyde (Sigma-Aldrich Chemie, Taufkirchen, Germany). After a PBS washing step, cells were resuspended in a small volume and drops of the cell suspension were incubated for 10 min on adhesion slides (Squarix biotechnology; Marl, Germany). Cells were then fixed with ice-cold methanol for 5 min in −20°C and then washed 3 times with PBS. After a 30 min blocking step with PBS containing 10% donkey-serum (Jackson Immuno Research Laboratories, West Grove, PA) primary antibodies anti-flotillin-1 (1∶150), anti-flotillin-2/ESA (1∶1000; both BD Pharmingen), anti-CD43-FITC (1G10; BD PharMingen), anti-CD44–FITC (J173; Coulter Immunotech, Krefeld, Germany), anti-moesin (1∶500; mAb 38/87; kindly provided by R.Schwartz-Albiez, Heidelberg) or mouse-anti-β-actin (1∶2500; AC-15; Sigma, Taufkirchen, Germany) were added in PBS/5% donkey-serum for 45 min at room temperature, washed and counterstained with Cy3/Alexa488–conjugated secondary antibodies (1∶200; Jackson Immuno Research) for 30 min at room temperature. Finally the labeled cells were washed thrice with PBS, and mounted in 75% glycerin containing propyl gallate (50 mg/mL) and DAPI (4,6 diamidino-2- phenylindole; 200 ng/mL; Roche, Mannheim, Germany). Cells were observed with an Axioplan 2 fluorescence microscope (Carl Zeiss, Goettingen, Germany) using a 100x/1.30 oil immersion objective. Cytochalasin B (Sigma-Aldrich Chemie GmbH) to destabilize actin was used at 10 µg/mL.

### Transfection of CD34^+^ Cells

Freshly isolated CD34^+^ cells were transfected with 5 µg Flotillin-2-EGFP-N1 or pEGFP-N1 (BD Clontech, Heidelberg, Germany) vector as a control using the nucleofection method (Amaxa, Cologne, Germany). On day 1 after transfection, living transfected cells were observed in time-lapse movies with an Axiocam digital camera in an Axioplan 2 fluorescence microscope (Carl Zeiss, Goettingen, Germany) using a 100x oil immersion objective. Time-lapse movies were processed using Axiovision 4.4 Software (Carl Zeiss).

### Cultivation and Differentiation of Monocytes

Peripheral blood mononuclear cells (PBMC) were prepared in cell preparation tubes (Vacutainer CPT, sodium citrate, BD Biosciences, Heidelberg, Germany) according to the manufacturer's instructions. After centrifugation (20 min, 1650 g) the white layer above the gel containing the PBMC was removed and the cells were washed three times with RPMI 1640 (BioWhittaker, Verviers, Belgium) containing 2.5 IU/ml heparin (Liquemin®, Hoffmann LaRoche, Grenzach-Wyhhylen, Germany). Cells were resuspended in RPMI 1640+100 IU/ml penicillin/streptomycin (BioWhittaker) +10% heat-inactivated FCS (Linaris, Wertheim-Bettingen, Germany), plated on Falcon Culture Slides (BD Biosciences) and incubated at 37°C and 5% CO_2_ for various durations. Some samples were additionally stimulated with 100 ng/ml recombinant human GM-CSF (Leucomax 300, Sandoz, Basel, Switzerland) at the start of incubation. After the incubation the lymphocytes were removed by washing the culture slides three times with PBS. The adherent monocyte-derived macrophages were subsequently fixed in methanol for 5 min at −20°C or 4% PFA/PBS for 20 min at room temperature and processed for immunofluorescence.

### Laser Scanning Confocal Microscopy of Peripheral Blood T Lymphocytes and Jurkat T Cells

After fixation, cells were processed for microscopy as described above in the immunostaining section. After 24 h, the slides were analyzed using an LSM 510 image browser software (LSM 510; Zeiss, Oberkochen, Germany) equipped with a Plan-Apochromat 100x oil immersion lens.

### Immunological Synapse (IS)

EBV-B 221 cells were suspended at 10^7^ cells/ml and incubated (or not) with 1 µg/ml of Staphylococcal Enterotoxin E (SEE; Toxin Technology) for 2 hours at 37°C, mixing every 20 min. Pulsed cells were washed and incubated for 15 min at 37°C with equal numbers of Flotillin-2-EGFP-N1-expressing T cells. Cells were then adhered to microscope slides coated with 50 mg/ml poly-L-lysine, fixed with 2% paraformaldehyde and mounted in 2.5% 1,4-diazobicyclo[2.2.2]octane (DABCO, Fluka), 90% glycerol, 10% PBS. Confocal microscopy was performed with a Biorad confocal microscope (BioRad), using laser excitation at 488 nm. Images were analyzed using Adobe Photoshop 7.0 and NIH-Image J programs. To quantify flotillin-2 recruitment at the IS, boxes were drawn at the IS, at cell membrane not in contact with the APC, and a background area outside the cell. The relative recruitment index (RRI) was calculated as indicated: [mean fluorescence intensity (MFI) at synapse – background]/[MFI at regions not in contact with APC – background].

### Two Photon Microscopy

Cells were labeled with 5 µM Laurdan (Molecular Probes) for 30 min at 37°C and imaged and analyzed as previously described [24], [25]. In brief, labeled cells were fixed with 4% paraformaldehyde and images obtained with a Leica DM IRE2 microscope [66], [67]. Laurdan was excited at 800 nm and emission intensities simultaneously recorded in the range of 400–460 nm and 470–530 nm. Intensity images were converted into Generalized Polarization (GP) images (WiT software) with
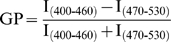
.

Final GP images were pseudo-colored in Adobe Photoshop. To determine GP values at the synapses, the region of interest (ROI) was defined as the membranes adjacent to the bead and the mean GP area of the ROI determined. GP values were corrected using the G-factor obtained for Laurdan in DMSO for each experiment [24]. For confocal images a helium-neon laser was used to excite Cy3 (Ex: 543 nm, Em: 550–620 nm) and obtain transmission (Ex: 633 nm). For all images a 100x oil objective, NA = 1.4 was used.

### Isolation of Detergent Resistant Membranes (DRMs) and Western Blotting

DRMs or the Triton X-100 resistant floating fractions of the T lymphocytes were isolated and western blotting of the fractions or lysates was performed essentially as described[23].

### Immunostaining of Filter Grown MDCK Cells

Immunostaining of filter-grown MDCK cells and microscopic examination were done essentially as described [68].

## Supporting Information

Video S1 Flotillin-2-GFP accumulates at uropod in transfected hematopoietic cells. Live imaging of Flotillin-2-GFP transfected-CD34 positive hematopoietic cells was done as described in the Materials and Methods section. Note that the flotillin domains are clustered at the uropod tip of the migrating cell.
(0.34 MB AVI)
